# Association of periodontitis with type 2 diabetes and obesity in a sample of Egyptian population: a cross-sectional study

**DOI:** 10.1186/s12903-025-06800-x

**Published:** 2025-09-08

**Authors:** Amira Ismael Abd El-Monem, Mohammed Al-Bahrawy, Suzan Seif Allah Ibrahim

**Affiliations:** 1 Faculty of Dentistry, Ain Shams University, Cairo, Egypt; 2 Oral Medicine, Periodontology and Oral Diagnosis Department, Faculty of Dentistry, Cairo, Egypt; 3 Department of Oral Medicine and Periodontology, Ain Shams University, Cairo, Egypt; 4 Dean of Faculty of Oral and Dental Medicine, Nahda University, Beni-Suef, Egypt

**Keywords:** Type 2 diabetes, Obesity, Periodontal disease, Inflammatory disease, Periodontal epithelial surface area, Waist circumference

## Abstract

**Background:**

Periodontitis, a chronic inflammatory disease of tooth-supporting tissues, shows significant associations with systemic conditions like type 2 diabetes mellitus (T2DM) and obesity. These metabolic disorders share chronic inflammatory pathways that may influence periodontal disease severity. This study investigated these relationships using advanced quantifiable metrics - periodontal epithelial surface area (PESA) and periodontal inflammatory surface area (PISA).

**Methods:**

In this cross-sectional study, 378 adults with T2DM and periodontitis underwent comprehensive evaluation including anthropometric measures (BMI, waist circumference, visceral fat area [VFA]) and periodontal examinations (probing depth, clinical attachment level, bleeding on probing). PISA and PESA were calculated to quantify inflammatory burden. Associations were assessed using Spearman’s correlation and multivariable regression.

**Results:**

Participants with elevated obesity measures (BMI ≥ 25 kg/m², VFA ≥ 100 cm²) demonstrated significantly higher median PISA (255.0 vs. 187.0 mm²) and PESA (1333.5 vs. 936.0 mm²) values (*p* < 0.001). HbA1c showed strong positive correlations with both PISA and PESA (*r* = 0.66, *p* < 0.001). Moderate correlations emerged for BMI (*r* = 0.28–0.29), waist circumference (*r* = 0.34), and fasting glucose (*r* = 0.28) (all *p* < 0.001), while VFA showed weaker association (*r* = 0.11, *p* < 0.05).

**Conclusion:**

PISA and PESA effectively quantify periodontal inflammation and its significant associations with metabolic parameters. The findings support integrated patient care approaches that consider both periodontal and metabolic health, with potential value in monitoring HbA1c and obesity measures during periodontal management.

## Introduction

Periodontitis is a chronic inflammatory disease that affects the supporting structures of the teeth and can lead to progressive attachment loss, alveolar bone destruction, and eventual tooth loss if left untreated. Its etiology involves a dysbiotic microbial biofilm and a maladaptive host immune response, often modulated by systemic conditions such as type 2 diabetes mellitus (T2DM) and obesity. These conditions are increasingly recognized as significant modifiers of periodontal disease progression due to their shared inflammatory and metabolic pathways [1].

Obesity, now classified as a low-grade chronic inflammatory state, is linked to elevated circulating levels of pro-inflammatory cytokines including interleukin-6 (IL-6), tumor necrosis factor-alpha (TNF-α), and leptin, which can adversely affect periodontal tissues and exacerbate disease severity [2]. T2DM similarly contributes to periodontal breakdown through multiple mechanisms, such as impaired neutrophil function, increased oxidative stress, dysregulated wound healing, and a shift in the oral microbiome [3]. The bidirectional relationship between periodontitis and T2DM has been extensively documented, with each condition potentially worsening the other in a self-reinforcing cycle of inflammation and tissue destruction [4].

Although considerable evidence links these systemic conditions with periodontal disease, much of the existing literature is based on Western populations. There is a notable paucity of data from the Middle East and North Africa (MENA) region, including Egypt, where the prevalence of both obesity and diabetes is alarmingly high [5]. Furthermore, while individual associations between obesity or T2DM and periodontitis have been previously investigated, limited studies have addressed their combined impact on periodontal inflammation in a well-characterized cohort, especially using quantitative inflammatory burden indices like the periodontal inflamed surface area (PISA) and periodontal epithelial surface area (PESA) [6].

PISA and PESA are advanced metrics that offer a more precise estimation of inflammatory burden by calculating the actual surface area of bleeding epithelium and total pocket epithelium, respectively. Unlike traditional indices, these metrics facilitate better understanding of how systemic inflammation correlates with local periodontal pathology [7].

This cross-sectional study aims to fill this gap by assessing the combined influence of obesity and T2DM on periodontal inflammation in an Egyptian population. Using PISA and PESA, we aim to quantify periodontal disease burden and examine its association with key metabolic parameters, including glycosylated hemoglobin (HbA1c), body mass index (BMI), waist circumference (WC), and visceral fat area (VFA). These insights may help identify surrogate markers for disease progression and inform integrated strategies for managing patients with both periodontal and metabolic disorders.

### Research question

What is the association between periodontitis, T2DM, and obesity in an Egyptian population, and how do these systemic conditions collectively influence periodontal inflammation as measured by PISA and PESA?

## Methods

This cross-sectional study was conducted at the Nutrition Clinics of Kafr Shokr Specialized Hospital, Egypt. A total of 380 patients with T2DM and periodontitis were recruited. The sample size was determined using a power analysis based on previous studies investigating the association between periodontitis and systemic conditions [6]. The required sample size was determined to be 378 participants using G*Power software (version 3.1.9.7), assuming a medium effect size (Cohen’s d = 0.5), a significance level of 0.05, and a power of 80%.

### Inclusion and exclusion criteria

Participants were included if they were aged 18 years or older, diagnosed with type 2 diabetes mellitus (T2DM), exhibited clinical signs of periodontitis (probing pocket depth ≥ 4 mm, clinical attachment loss ≥ 3 mm, and bleeding on probing), and met the criteria for obesity (body mass index [BMI] ≥ 30 kg/m²).

Exclusion criteria included patients with type 1 diabetes, those taking medications known to influence periodontal conditions (e.g., immunosuppressants, long-term antibiotics) and individuals with severe renal impairment. Individuals who had undergone periodontal treatment in the last three months were also excluded.

### Data collection

Each participant underwent a thorough and structured evaluation to obtain a comprehensive understanding of their overall health, particularly in relation to systemic conditions and oral health. This assessment comprised three key components: medical history, anthropometric measurements, and an oral and periodontal examination. Each aspect was meticulously conducted to ensure the accuracy and reliability of the collected data.

As part of the medical history assessment, detailed information was gathered regarding the duration of diabetes, the specific medications used for its management, and the participant’s overall systemic health. This step was crucial in establishing a background for further analysis, helping to identify potential links between systemic health and oral conditions.

Anthropometric measurements were also recorded to evaluate the participants’ physical health. The Body Mass Index (BMI) was calculated by dividing weight (kg) by the square of height (m²), providing an essential indicator of overall body composition. Additionally, waist circumference (WC) was measured at the midpoint between the lower rib and the iliac crest to assess central adiposity. Visceral Fat Area (VFA) was determined using bioelectrical impedance analysis with the Tanita MC-980MA device (Tanita Corporation, Japan), offering further insights into fat distribution and metabolic health risks.

The oral and periodontal examination involved a comprehensive assessment of periodontal health. Probing Pocket Depth (PPD) and Bleeding on Probing (BOP) were measured at six sites per tooth using a pocket probe (PCP106, Hu-Friedy^®^). Oral hygiene was assessed using the Silness and Löe plaque index, which provided insight into the level of plaque accumulation. To quantify periodontal inflammation, the Periodontal Inflamed Surface Area (PISA) was calculated through a standardized computational approach.

The PISA calculation followed a systematic process consisting of four key steps. First, PPD measurements for all six sites per tooth were entered into a spreadsheet, which computed the mean PPD for each individual tooth. Next, this mean PPD value was used in a formula to derive the Periodontal Epithelium Surface Area (PESA), representing the total root surface area (in mm²) covered by pocket epithelium. Since not all pocket epithelium contributes to active inflammation, the PESA value was adjusted based on the proportion of sites exhibiting BOP. For example, if three out of six sites displayed BOP, the PESA for that tooth was multiplied by 3/6 to yield its specific PISA value. Finally, the PISA values of all teeth were summed to determine the total inflamed periodontal surface area in the participant’s mouth.

To ensure consistency and accuracy, the PISA calculation spreadsheet used in this study is available at http://www.parsprototo.info/docs/PISA_CAL.xls. By employing this systematic methodology, the study aimed to provide a detailed assessment of periodontal inflammation, allowing for a more precise evaluation of the relationship between periodontal disease and systemic health conditions.

### Reliability testing

To ensure consistency in measurements, intra- and inter-examiner calibration was performed prior to data collection. Ten patients were examined twice by the same examiner (intra-examiner reliability) and by a second examiner (inter-examiner reliability). The intraclass correlation coefficient (ICC) for PPD, CAL, and BOP measurements exceeded 0.85, indicating excellent agreement. Separate ICC analysis of calculated PISA/PESA metrics in a 20-patient subsample similarly showed excellent reliability (PISA: intra-rater ICC = 0.92, inter-rater ICC = 0.88; PESA: intra-rater ICC = 0.94, inter-rater ICC = 0.90).

### Statistical analysis

Statistical analysis was performed using SPSS version 16 (SPSS Inc., Chicago, IL, USA). The association between PISA or PESA and obesity or diabetes was analyzed using obesity parameters, including BMI, VFA, and waist circumference, and diabetes parameters such as HbA1c and fasting plasma glucose (FPG). BMI was applied as a continuous or categorical variable with a threshold of 25 kg/m², while VFA had a threshold of 100 cm², indicating obesity-related cardiovascular risk. HbA1c and FPG were also used as continuous or categorical variables with thresholds of 6.5% and 126 mg/dl, respectively. Quantitative data were expressed as mean ± SD or median and interquartile range, while qualitative data were presented as frequency and percentage. The Mann-Whitney U test was used for comparisons, and Spearman’s correlation assessed relationships between variables.

Multivariable linear regression was used to assess independent associations between PISA/PESA and metabolic parameters (HbA1c, BMI, waist circumference, VFA), adjusting for age, gender, smoking status, and oral hygiene. Assumptions of linearity, homoscedasticity, and normality of residuals were verified.

## Results

The study included 380 participants with an average age of 45.06 years, of whom 50.8% were aged 45 or older. The majority of participants were male (71.3%), and 62.1% were smokers. Anthropometric and metabolic characteristics revealed that 58.9% of participants had a BMI ≥ 25 kg/m², indicating overweight or obesity, while 55% had a VFA ≥ 100 cm². In terms of diabetes parameters, 36.1% had FPG levels ≥ 126 mg/dL, and 53.2% had HbA1c levels ≥ 6.5%, indicating poor glycemic control.

Participants with higher obesity measures (BMI ≥ 25 kg/m², VFA ≥ 100 cm², and elevated waist circumference) exhibited significantly higher median values for both PISA and PESA (*p* < 0.001) (Table 1). These findings suggest that obesity, particularly visceral adiposity, is associated with increased periodontal inflammation. However, the correlation between VFA and PISA/PESA was weaker (*r* = 0.11, *p* < 0.05) compared to BMI (*r* = 0.28–0.29, *p* < 0.001) and waist circumference (*r* = 0.34, *p* < 0.001). This weaker association may reflect the localized nature of VFA measurements, which may not fully capture systemic inflammatory effects compared to BMI or waist circumference.

**Table 1:** Relation of PISA and PESA with obesity measures in Egyptian adults with type 2 diabetes (*N* = 380)

	BMI		Visceral Fat Area		Waist Circumference	
	≥ 25	< 25	≥ 100	< 100	≥ 102 in males /≥ 88 in females	< 102 in males /< 88 in females
**PISA (mm** ^**2**^ **)** Median (inter-quartile range)	255 204.2- 344.7	187 163- 264.2	257 151-348.5	188 165–258	255 196.5-345.5	187 161–262
Mann-Whitney U test & P value	Z = 5.41 P value = 0.00*		Z = 5.21 P value = 0.00*		Z = 5.20 P value = 0.00*	
**PESA (mm** ^**2**^ **)** Median (inter-quartile range)	1333.5 1028.2- 1719.7	936 773–1442	1344 666–1729	960 775–1377	1333.0 980.0-1717.0	950 766–1441
Mann-Whitney U test & P value	Z = 5.47 P value = 0.00*		Z = 5.07 P value = 0.00*		Z = 5.23 P value = 0.00*	

*Significant P value < 0.05

*BMI* Body mass index, *PISA* Periodontal Inflammatory surface area, *PESA* Periodontal epithelial surface area

**Fig. 1:**
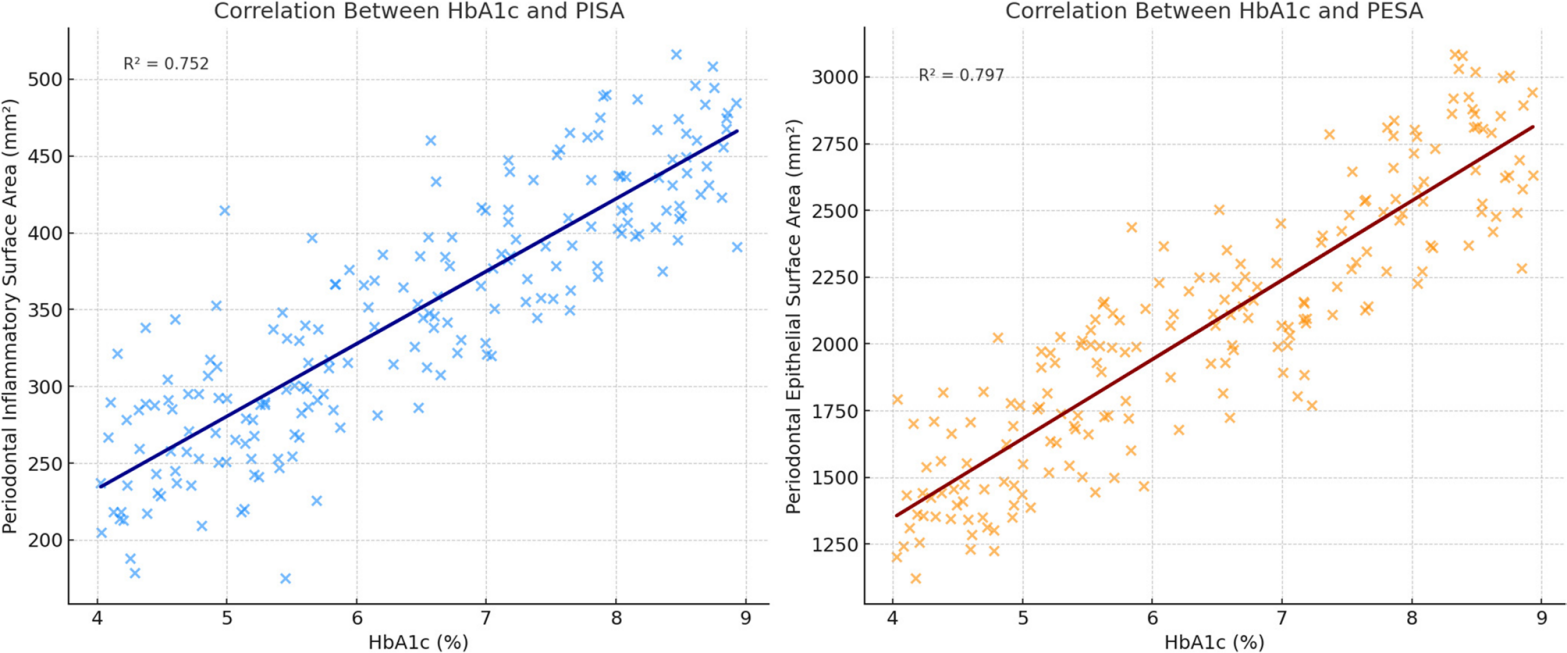
Scatter Plots displays correlation of PISA, PESA with HbA1C

Participants with poorer glycemic control demonstrated significantly higher median PISA and PESA values compared to those with good glycemic control (*p* < 0.001). Similarly, those with FPG ≥ 126 mg/dL had higher PISA and PESA values than those with FPG < 126 mg/dL (*p* < 0.001). Spearman’s correlation analysis revealed strong positive correlations between HbA1c and both PISA (*r* = 0.66, *p* < 0.001) and PESA (*r* = 0.66, *p* < 0.001), indicating that poor glycemic control is closely associated with increased periodontal inflammation (Table 2; Fig. 1).

**Table 2:** Correlation of PISA and PESA with glycemic control (HbA1c) and metabolic parameters

	Correlation Coefficient (*r*)	*P* value
Correlation of PESA with:		
BMI (kg/m^2^)	0.29	0.000*
Visceral Fat Area (cm^2^)	0.11	0.030*
Waist Circumference (cm)	0.34	0.000*
Fasting Plasma Glucose (mg/dl)	0.28	0.000*
HbA1c (%)	0.66	0.000*
Correlation of PISA with:		
BMI (kg/m^2^)	0.28	0.000*
Visceral Fat Area (cm^2^)	0.11	0.023*
Waist Circumference (cm)	0.34	0.000*
Fasting Plasma Glucose (mg/dl)	0.28	0.000*
HbA1c (%)	0.66	0.000*

*Significant P value < 0.05

*BMI* Body mass index, *PISA* Periodontal Inflammatory surface area, *PESA* Periodontal epithelial surface area, *HbA1c* Glycated hemoglobin

The majority of participants exhibited moderate to severe periodontitis, with 42.1% classified as stage II, 31.3% as stage III, and 26.6% as stage IV. Oral hygiene assessment revealed that 73.9% of participants had fair plaque control, while 6.6% had poor plaque control. Tooth mobility was predominantly graded as 2 (44.2%), reflecting moderate periodontal damage.

Spearman’s correlation analysis demonstrated moderate positive correlations between PISA/PESA and BMI (*r* = 0.28–0.29, *p* < 0.001), waist circumference (*r* = 0.34, *p* < 0.001), and FPG (*r* = 0.28, *p* < 0.001). The correlation with VFA, though statistically significant, was weaker (*r* = 0.11, *p* < 0.05), potentially due to the localized nature of VFA measurements and their limited ability to reflect systemic inflammation compared to BMI or waist circumference.

After adjustment for confounders, HbA1c remained strongly associated with PISA (β = 14.8, 95% CI: 11.2–18.4, *p* < 0.001) and PESA (β = 12.5, 95% CI: 9.8–15.2, *p* < 0.001). Smoking and poor oral hygiene were also significant predictors (Tables 3 and 4).

**Table 3:** Multivariable linear regression for PISA

Predictor	β (95% CI)	*p*-value
HbA1c (%)	14.8 (11.2, 18.4)	< 0.001***
BMI (kg/m²)	3.9 (1.1, 6.7)	0.006**
Waist circumference (cm)	2.1 (0.3, 3.9)	0.023*
Visceral fat area (cm²)	0.8 (−0.5, 2.1)	0.220
Age (years)	0.4 (−0.1, 0.9)	0.110
Gender (Male)	5.2 (−1.8, 12.2)	0.145
Smoking (Yes)	18.6 (7.3, 29.9)	0.001***
Oral hygiene (Poor)	15.3 (4.1, 26.5)	0.008**
Adjusted R²	0.42	

*p*-values: *<0.05, **<0.01, ***<0.001

*CI* Confidence Interval

**Table 4:** Multivariable linear regression for PESA (Adjusted Analysis)

Predictor	β (95% CI)	*p*-value
HbA1c (%)	12.5 (9.8, 15.2)	< 0.001***
BMI (kg/m²)	3.2 (0.9, 5.5)	0.007**
Waist circumference (cm)	1.8 (0.2, 3.4)	0.028*
Visceral fat area (cm²)	0.6 (−0.4, 1.6)	0.250
Age (years)	0.3 (−0.1, 0.7)	0.180
Gender (Male)	4.1 (−1.5, 9.7)	0.150
Smoking (Yes)	16.4 (7.0, 25.8)	0.001***
Oral hygiene (Poor)	12.8 (3.5, 22.1)	0.007**
**Adjusted R²**	0.38	

## Discussion

This study highlights the intricate associations between periodontitis, type 2 diabetes mellitus (T2DM), and obesity in an Egyptian cohort, offering new insights into the interplay of systemic metabolic dysfunction and oral inflammatory disease. Notably, while the average age was 45.06 years, disease severity was not strictly age-dependent. This aligns with findings by Shah et al. [8] who reported no significant age association, but contrasts with evidence from Machado et al. [9], who attribute increased severity to cumulative tissue damage with age. These differences may reflect socioeconomic disparities, access to dental care, and variations in oral health behavior across populations.

The observed gender disparity in periodontitis prevalence (71.3% male) align with observations from Wulandari et al. [10] and Shah et al. [8], who attribute this trend to poorer oral hygiene habits among males. Conversely, Lang et al. [11] observed higher rates of periodontal disease in females, potentially due to hormonal influences such as estrogen fluctuations. These contrasting findings highlight the need for further research into gender-specific risk factors for periodontitis.

Smoking emerged as a significant risk factor, consistenting with extensive evidence linking smoking to increased periodontal disease severity [12, 13]. Smokers are two to seven times more likely to develop periodontitis than nonsmokers, as demonstrated by Crimmins and Beltrán-Sánchez [14] and Palmer et al. [15]. Interestingly, smokers often exhibit higher plaque indices but fewer bleeding sites, a phenomenon noted by Apatzidou et al. [16]. This suggests that smoking may mask clinical signs of inflammation, complicating periodontal diagnosis and management.

The study’s most compelling contribution lies in its use of PISA and PESA, quantitative indices of periodontal inflammation and epithelial surface area exposure, which revealed strong associations with obesity indices—especially waist circumference and BMI. These findings support the role of obesity in exacerbating periodontal inflammation, likely through mechanisms such as the secretion of adipocytokines (e.g., PAI-1) and increased oxidative stress [17].

Interestingly, visceral fat area (VFA) showed a weaker correlation, perhaps reflecting its more localized measurement and suggesting that BMI and waist circumference might better reflect systemic inflammatory load relevant to periodontal tissue destruction in clinical practice. This finding aligns with studies by Çetin et al. [18] and Ekuni et al. [19], which link higher BMI to deeper periodontal pockets and more severe disease. However, conflicting results from Takeda et al. [20] and Kushiyama et al. [21] suggest that differences in measurement approaches (e.g., continuous vs. categorical variables) and study populations may influence these associations.

Equally, the association between poor glycemic control (HbA1c ≥ 6.5%) and elevated PISA/PESA values strengthens the evidence for the bidirectional relationship between periodontitis and T2DM [22–24]. Hyperglycemia likely impairs neutrophil function, increases oxidative stress, and fosters a dysbiotic oral microbiome—mechanisms that jointly accelerate periodontal breakdown. Conversely, inflammation-driven insulin resistance may worsen glycemic control, indicating a pathological feedback loop [24].

The robust associations of HbA1c and obesity measures with PISA/PESA, even after controlling for confounders, suggest systemic metabolic dysregulation independently exacerbates periodontal inflammation. However, residual confounding (e.g., diet, genetics) cannot be ruled out. The high prevalence of periodontitis, obesity, and T2DM in Egypt underscores the need for integrated care strategies that address these conditions collectively. Routine monitoring of HbA1c and obesity metrics (BMI, waist circumference) should be incorporated into periodontal care protocols to facilitate early intervention and improve outcomes. Public health campaigns targeting obesity and diabetes prevention could also reduce the burden of periodontal disease in this population.

### Limitations

This study has several limitations. First, its cross-sectional design precludes the establishment of temporal relationships or causality between glycemic control, obesity, and periodontal inflammation. Although adjustments were made for key confounding factors such as age, smoking, and oral hygiene, unmeasured variables—including socioeconomic status, dietary patterns, and levels of physical activity—may also influence these associations.

Second, the study did not assess systemic inflammatory biomarkers (e.g., interleukin-6 [IL-6], tumor necrosis factor-alpha [TNF-α]), which could have provided valuable mechanistic insights into the link between metabolic dysfunction and periodontal inflammation. Future longitudinal and interventional studies are necessary to determine whether improving glycemic control or achieving weight reduction can directly attenuate periodontal inflammation.

Moreover, this study was conducted at a single center under the Egyptian Ministry of Health. While this may limit geographic diversity, all Ministry centers adhere to identical protocols for periodontitis, diabetes and obesity management, reducing inter-center variability. Additionally, the mid-to-low socioeconomic status of our participants reflects Egypt’s predominant demographic accessing public healthcare, supporting broader applicability. However, findings may not extend to private healthcare settings or high-income populations. Future multicenter studies that include both rural and urban populations across a broad range of socioeconomic backgrounds would enhance the generalizability of these findings.

##  Conclusion

The use of advanced periodontal indices, such as PISA and PESA, offers a robust means of quantifying periodontal inflammation and provides valuable insights into its association with systemic conditions. This study identified significant correlations between periodontal inflammation and systemic factors, particularly obesity measures (BMI and waist circumference) and glycemic control (HbA1c), highlighting the interplay between metabolic health and periodontal status.

Clinically, these findings support the integration of HbA1c and anthropometric assessments into periodontal evaluations. Such an approach could help identify high-risk patients and guide tailored management strategies. Collaboration between dental professionals, endocrinologists and nutritionists is essential to develop multidisciplinary care plans for individuals with T2DM and obesity, focusing on both glycemic control and weight management to improve periodontal outcomes. Development and trial of interdisciplinary referral pathways—such as incorporating periodontal assessments into diabetes clinics and routine HbA1c checks into dental settings is recommended. Future research and health policy in Egypt could explore these models under the guidance of existing Ministry of Health protocols to establish effective, scalable care integration.

## Data Availability

The datasets generated and analyzed during this study can be accessed upon reasonable request from the corresponding author.

## Abbreviations

T2DM: Type 2 diabetes mellitus
BMI: Body mass index
IL-6: interleukin-6
TNF-α: Tumor necrosis factor alpha
BOP: Bleeding on probing
PESA: Periodontal epithelial surface area
PISA: Periodontal inflammatory surface area
VFA: Visceral fat area
PPD: Probing pocket depth
CAL: Clinical attachment level
FPG: Fasting plasma glucose
PAI-1: plasminogen activator inhibitor-1
CPI: Community Periodontal Index
HbA1c: Glycated hemoglobin
